# Protective Role of Chronic Exercise Training in Modulating the Impact of Hyperglycemia on Vascular Sensitivity to Ischemia-Reperfusion

**DOI:** 10.3390/nu15010212

**Published:** 2023-01-01

**Authors:** Antoine Grandperrin, Mathilde Bourgoin, Sandrine Gayrard, Doria Boulghobra, Guillaume Walther, Cyril Reboul, Grégory Meyer

**Affiliations:** Laboratoire de Physiologie Expérimentale Cardiovasculaire (UPR-4278, LaPEC), Avignon University, F-84000 Avignon, France

**Keywords:** exercise, vascular function, hyperglycemia, ischemia reperfusion

## Abstract

Hyperglycemia (HG) is associated with increased mortality and morbidity in acute ischemic events. Regardless of the tissue or organs involved, the vascular endothelium is a key target of ischemia-reperfusion (I/R) injury severity. Among endothelium-protective strategies, exercise has been widely described as useful. However, whether this strategy is able to impact the deleterious effect of HG on endothelial function during I/R has never been challenged. For this, 48 male Wistar rats were randomized into 4 groups: sedentary (Sed) or exercised (Ex, 45 min/day, 5 days/week for 5 weeks) rats, treated (hyperglycemic, HG) or not (normoglycemic, NG) with streptozotocin (40 mg/kg, 48 h before procedure). Vascular I/R (120/15 min) was performed by clamping the femoral artery. Arterial and downstream muscular perfusions were assessed using laser speckle contrast imaging. Vascular endothelial function was assessed in vivo 15 min after reperfusion. HG was responsible for impairment of reperfusion blood flow as well as endothelial function. Interestingly exercise was able to prevent those impairments in the HG group. In agreement with the previous results, HG increased reactive oxygen species production and decreased nitric oxide bioavailability whereas exercise training normalized these parameters. It, therefore, appears that exercise may be an effective prevention strategy against the exacerbation of vascular and muscular damage by hyperglycemia during I/R.

## 1. Introduction

Hyperglycemia (HG) without preexisting diabetes mellitus is an established risk factor for increased mortality and morbidity after acute ischemic events [1,2]. Earlier studies have shown that high blood sugar levels upon admission to the hospital are common in patients experiencing acute ischemic events, such as heart attack or stroke. These high blood sugar levels have been linked to an increased risk of death and complications during hospitalization [3,4,5,6]. Previous studies from our team and others have provided evidence that HG transiently impairs endothelial function [7,8]. These alterations seem to be strongly dependent on the alteration of the nitric oxide (NO) pathway as a consequence of increased oxidative stress [9,10,11,12]. During ischemia-reperfusion (I/R), HG was reported to impair vascular function during reperfusion [13,14]. Indeed, upon reperfusion, abrupt oxygen delivery to ischemic arteries results in a sharp increase in the production of reactive oxygen species (ROS) at the level of the endothelium [15]. This phenomenon is exacerbated in hyperglycemic conditions. Indeed, previous studies have observed an increase in ROS production when I/R was performed in HG [13,14]. The subsequent increase in oxidative stress is responsible for a decrease in NO availability associated with endothelial dysfunction [14].

Consequently, there is considerable interest in studying prevention strategies that would reduce vascular sensitivity to ischemic pathologies in the context of HG. Yet, exercise training was proven to be able to limit endothelial vulnerability to I/R in both human [16,17] and rodent models [18,19]. Indeed, exercise training has been reported to attenuate I/R impairments by improving NO bioavailability and lowering oxidative stress and inflammation [20,21]. However, to our knowledge, despite the large number of studies investigating the effect of exercise training on vascular I/R, the protective effect of chronic exercise in minimizing vascular susceptibility to I/R damage under HG conditions has not been studied. In this context, this study aimed to investigate whether exercise training was able to protect the vascular endothelium from reperfusion injuries in a rat model of HG.

In light of the beneficial effects of exercise training on I/R damage reported in the literature, we hypothesized that exercise training would reduce the increased vascular sensitivity to I/R observed in HG. Moreover, since exercise training was previously reported to improve vascular function through an enhanced balance between ROS production and NO availability, our second hypothesis was that such vascular protection would be the consequence of a preserved endothelial function dependent on decreased oxidative stress.

To investigate these hypotheses, laser speckle contrast imaging was used to study the effects of chronic exercise and HG on vascular sensitivity to I/R in the femoral artery. The main result of our work was that chronic exercise was able to protect vascular function from HG-dependent reperfusion impairments. These preventive effects of exercise training in the HG condition appear to be the consequence of a reduced production of ROS associated with increased NO availability.

## 2. Methods

### 2.1. Population and Experimental Design

Six-week-old male Wistar rats (*n* = 48) were purchased from Janvier Labs (Le Genest-Saint-Isle, France). They were kept in cages with an enriched environment that maintained controlled environmental conditions, and a 12 h light/dark cycle. All procedures were performed in accordance with the local research ethics committee (CEEA n°14) and the agreement of European and French Ministry of Agriculture about the care and use of laboratory animals 2010/63/EU (APAFIS #33264-202109161036314). Rats were fed water and food ad libitum with a normal chow diet (A04, SAFE, France).

After 2 weeks of acclimatization, rats were randomly assigned into two groups: sedentary (Sed; *n* = 24) or exercised (Ex; *n* = 24) rats. First, rats from the exercised group were familiarized with the motorized treadmill by running at 15 m.min^−1^ for 15 min daily for a week. Afterward, rats were trained at 60% of their expected maximal aerobic velocity (25 m·min^−1^) for 45 min per day, five times a week for 5 weeks as previously described [22,23]. To obtain replicable hyperglycemia that was stable throughout the I/R protocol, both groups of rats were treated (HG-Sed and HG-Ex) or not (NG-Sed and NG-Ex) with streptozotocin (STZ) (I.P.; 40 mg·kg^−1^) 48 h before arterial I/R was performed. Indeed, this single STZ injection was carried out as described in several previous studies to induce pancreatic islet destruction and induce a significant increase in blood glucose 48 h later [14,24,25]. The inclusion criterion for the HG groups was set at a blood glucose level above 200 mg·dL^−1^.

### 2.2. Femoral Artery Ischemia Reperfusion Protocol

To evaluate vascular sensitivity to I/R, we chose to perform an I/R protocol on the femoral artery to avoid confounding factors from cardiac or brain energetic metabolic activity. Indeed, the femoral artery is a convenient location for measuring blood flow in the arteries and only requires a superficial surgical procedure for access. Vascular perfusion measurements were implemented using laser speckle contrast imaging with a PeriCam PSI System (PeriMed, Stockholm, Sweden). To perform this, rats were anesthetized with isoflurane (4.5% induction; 1.5–2% maintenance), and constant body temperature (37 °C) was maintained by the use of a heating pad. An incision was made in the skin of the hind limbs directly above the femoral vasculature to expose both femoral arteries. A femoral artery was occluded right behind the inguinal ligament by using an arterial clamp to induce ischemia for 120 min. Then, the clamp was removed to induce reperfusion for 15 min. At the same time, a sham procedure was performed on the contralateral hind limb to standardize the data. A first region of interest (ROI) was placed in the distal part of the femoral artery just above the bifurcation with the popliteal artery of the ischemic and contralateral hind limb. To evaluate the consequence of vascular alteration on downstream tissues, a second region of interest was placed on the lower part of the quadriceps femoris muscle of both hind limbs. Experiments were performed at the same time of the day, with constant artificial light exposure and room temperature. The I/R protocol was performed by the same investigator throughout the protocol.

### 2.3. Vascular Reactivity

To evaluate the effect of HG and/or chronic exercise on vascular function following I/R, we subjected 6–8 rats from each group to an in vivo vascular reactivity challenge using topical administration of vasomotor drugs, as previously described in other vascular territories in previous studies [26,27,28,29,30,31]. To evaluate the effect of I/R in each group, dose–responses were performed on both the control artery (i.e., on the contralateral femoral arteries) and the ischemic artery (following the I/R protocol). First, phenylephrine (10^−5^ M, 50 µL) was topically applicated along the femoral artery to normalize vessel preconstriction. Then, a dose–response to acetylcholine (ACh, 10^−5^ M to 10^−2^ M, 50 µL) was performed, followed by a dose–response to phenylephrine (PE, 10^−6^ to 10^−2^ M, 50 µL). Each dose of ACh was applied for 5 min and each dose of PE for 3 min to evaluate the effect of HG and/or exercise on endothelium-dependent vasodilatation or smooth muscle-dependent vasoconstriction, respectively.

### 2.4. ROS and NO Quantification

To investigate the underlying mechanism of the endothelial dysfunction exhibited by our in vivo results, we used the ROS-and NO-sensitive dihydroethidium (DHE) and dye 4,5-diaminofluorescein diacetate (DAF-2), respectively, to evaluate the impact of our experimental conditions on these parameters. After 15 min of reperfusion, femoral arteries were collected (*n*= 4–5 rats per group; 2 samples per rat). After removal of adherent tissues, arteries were cut into small segments (1 mm long), embedded in Optimal Cutting Temperature (OCT from Tissue-Tek), and flash-frozen in liquid nitrogen. Transverse serial cross-sections (14 µM) were obtained using a cryostat-maintained at −20 °C (Leica Microsystems, Blenheim, Germany). ROS production was evaluated using DHE. Frozen sections were covered with 100 µM DHE and incubated in a light-protected humidified chamber at 37 °C for 5 min. Fluorescence microscopy and digital image acquisition were carried out using an EVOS M5000 (Thermo Fischer Scientific, Waltham, Massachusetts, USA). NO production was evaluated using the NO-sensitive fluorescent DAF-2 (Sigma-Aldrich, Saint-Louis, MS, USA). The same protocol was performed except that femoral artery segments were incubated for 1 h with DAF-2 (10 µM) in a light-protected humidified chamber at 37 °C before inclusion in OCT. Both DHE and DAF-2 fluorescence intensity were quantified using ImageJ software (National Institutes of Health).

### 2.5. Statistics

Data are expressed as the mean ± SEM. For comparison of experimental conditions, an analysis of variance (ANOVA) or repeated measures ANOVA followed by Tukey’s adjusted post hoc test were used. Repeated measured ANOVA was used to analyze vascular contraction and endothelial-dependent relaxation values between groups. Values of *p* < 0.05 were considered statistically significant. Finally, standardized effect sizes (ES, Cohen’s d) were calculated by comparing differences between groups according to recommendations [32]. ES was calculated by dividing the difference between groups by the average of their standard deviations. Small ES range from 0 to 0.59, moderate ES range from 0.6 to 1.19, large range from 1.2 to 1.99, very large range from 2.0 to 3.99, and ES are considered extremely large over 4.0 [33]. Statistical analyses were performed with GraphPad Prism version 8.0.0 for Windows (GraphPad Software, San Diego, CA, USA).

## 3. Results

### 3.1. Impact of Hyperglycemia and Exercise Training on Vascular Function

**Figure 1:**
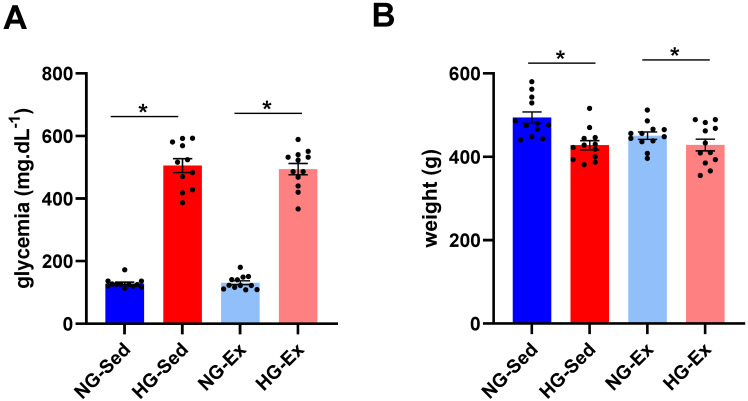
Impact of streptozotocin and exercise training on body weight and glycemia. Impact of hyperglycemia induced by streptozotocin (40 mg·kg^−1^ I.P., administered 48 h before ischemia-reperfusion) and exercise training (45 min per day, five times a week for 5 weeks) on (**A**) body weight and (**B**) glycemia of *n* = 12 rats per group. NG: normoglycemic; Sed: sedentary; HG: hyperglycemic; Ex: exercise training. *: *p* < 0.05. All values are expressed as the mean ± SEM. Comparison of multiple experimental conditions was performed using analysis of variance (ANOVA) followed by Tukey’s post hoc test.

In line with previous studies [24,25], we first confirmed that 48 h after STZ injection a marked increase in blood glycemia was observed in the HG groups (to the same extent in both groups; NG-Sed vs. HG-Sed, *p* < 0.001, ES = 7.05; NG-ex vs. HG-Ex, *p* < 0.001, ES = 7.73)) (Figure 1A). This increase was associated with a slight reduction in body weight (NG-Sed vs. HG-Sed, *p* = 0.002, ES = 1.52; NG-ex vs. HG-Ex, ES = 0.55) (Figure 1B).

**Figure 2:**
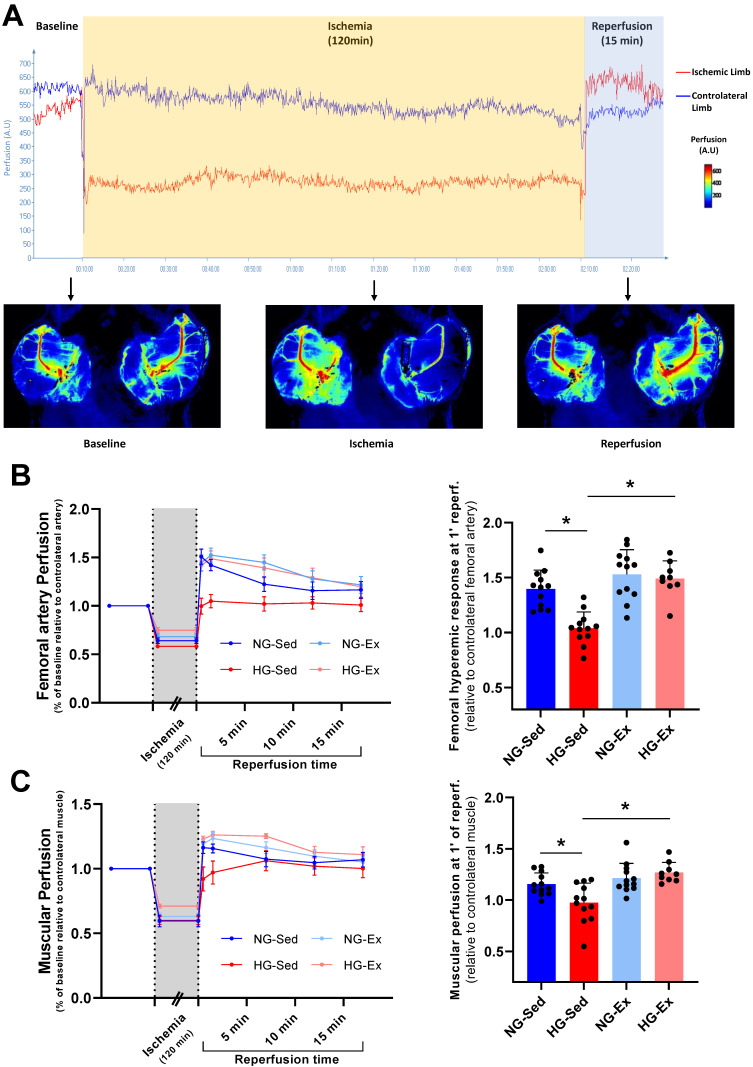
Beneficial impact of exercise training on the artery and downstream muscular perfusion in hyperglycemic conditions. (**A**) Experimental protocol of vascular ischemia-reperfusion (120 min./15 min.) using laser Speckle contrast imaging to evaluate arterial and downstream muscular perfusion levels. Perfusion was evaluated in the ischemic-limb (red) and the contralateral limb (blue). (**B**) Femoral artery perfusion kinetics evaluated using laser speckle contrast imaging during the ischemia-reperfusion protocol (left panel) and hyperemic response evaluated through vascular perfusion at 1 min of reperfusion (right panel). (**C**) Muscular perfusion during ischemia-reperfusion evaluated using laser speckle contrast imaging (left panel) and hyperemic response evaluated through muscular perfusion at 1 min of reperfusion (right panel). *n* = 10–12 rats per group. NG: normoglycemic; Sed: sedentary; HG: hyperglycemic; Ex: exercise training. *: *p* < 0.05. All values are expressed as the mean ± SEM. Comparisons of multiple experimental conditions were performed using analysis of variance (ANOVA) or repeated measures ANOVA followed by Tukey’s post hoc test.

**Figure 3:**
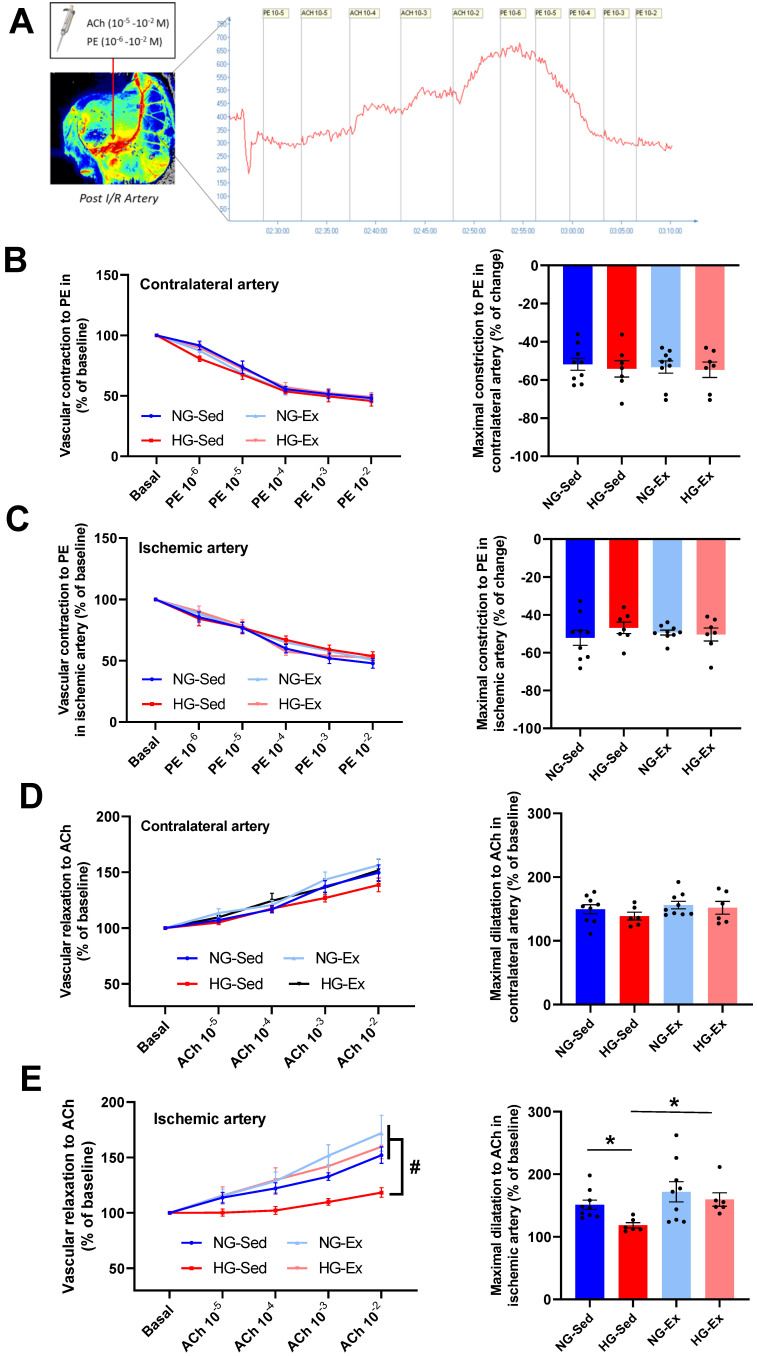
Beneficial impact of chronic exercise training on vascular reactivity in hyperglycemic conditions. (**A**) In vivo protocol of dose–response to acetylcholine (ACh, 10^−5^ to 10^−2^ M) and phenylephrine (PE 10^−6^ to 10^−2^) used to evaluate endothelial and smooth muscle function. Perfusion was evaluated using laser speckle contrast imaging. Vascular constriction to phenylephrine on the (**B**) control artery (contralateral limb) and (**C**) ischemic artery. Left panels represent the dose response to phenylephrine (10^−6^ to 10^−2^ M), and right panels represent the maximal constriction to phenylephrine. Vascular relaxation in response to acetylcholine in the (**D**) control artery (contralateral limb) and (**E**) ischemic artery. Left panels represent the dose response to acetylcholine (10^−5^ to 10^−2^ M), and right panels represent maximal relaxation to acetylcholine. *n* = 7–8 rats per group. ACh: acetylcholine; PE: phenylephrine; NG: normoglycemic; Sed: sedentary; HG: hyperglycemic; Ex: exercise training. *: *p* < 0.05; #: significantly different from HG-Sed (*p* < 0.05). All values are expressed as the mean ± SEM. Comparisons of multiple experimental conditions were performed using analysis of variance (ANOVA) or repeated measures ANOVA followed by Tukey’s post hoc test.

In NG, we reported that following 2 h of ischemia (Figure 2A), reperfusion was associated with a significant rise in arterial blood flow during early reperfusion [34,35,36,37]. Interestingly, this phenomenon was abolished when I/R was performed on HG animals (Figure 2B,C). Indeed, the perfusion peak expressed relative to the contralateral femoral artery observed consecutively to reperfusion was significantly lower in the femoral artery (Figure 2B, NG-Sed vs. HG-Sed, *p* = 0.003, ES = 2.16) and in the downstream muscle area (Figure 2C, NG-Sed vs. HG-Sed, *p* = 0.01, ES = 1.16) in the HG-Sed group than in the NG-Sed group. Considering that this rapid increase in arterial blood flow is mainly attributed in the literature to hyperemic endothelium-dependent vasodilation, we next evaluated the impact of our experimental conditions on the vascular response to vasoactive molecules in vivo (Figure 3A). We first reported that the vasoconstrictive responses to PE were not impacted by either HG (contralateral artery, Figure 3B) or I/R (ischemic artery, Figure 3C). However, in line with the results obtained, we reported that the response to ACh, known to activate endothelium-dependent vasodilation, was strongly impacted by I/R in HG animals. Indeed, no difference was reported on the contralateral hindlimb, highlighting that short-term HG has no marked impact on the vasorelaxation response to ACh (Figure 3D). In contrast, following I/R, the vascular response to ACh was markedly reduced in HG animals compared to NG animals (Figure 3E, NG-Sed vs. HG-Sed, *p* = 0.03, ES = 1.77). Altogether, these results contribute to showing that HG was responsible for a higher sensitivity of endothelial function to I/R.

Considering the deleterious impact of HG on vascular sensitivity to I/R, we next evaluated whether chronic exercise training constitutes a good strategy to prevent this phenomenon. In the exercised groups, we reported no major impact when I/R was performed in NG conditions since no change in reperfusion blood flow was observed (Figure 2B,C). However, in the HG group, exercise training was able to prevent reperfusion-induced vascular impairments. Indeed, at the time of reperfusion, previously trained rats from the HG group had higher vascular blood flow (HG-Ex vs. HG-Sed, *p* = 0.007, ES = 2.72) and subsequent downstream muscular perfusion values (HG-Ex vs. HG-Sed, *p* = 0.04, ES = 1.93) than their sedentary counterparts (Figure 2B,C). To determine whether these changes at reperfusion are dependent on the effects of exercise on vascular function, we next assessed endothelial and smooth muscle function in both arteries of different groups. No change in the vascular response to PE was reported in either the control or ischemic artery under NG or HG conditions. More surprisingly, no significant effect of training on the vasodilatory response to ACh was observed in the NG condition in either the control (+4% maximal response (Rmax), Figure 3D) or the ischemic (+13% Rmax, Figure 3E) artery. No effect of physical activity was reported in the HG condition on the contralateral artery (+5% Rmax; Figure 3D). However, a major finding of this work is that exercise was able to prevent exacerbation of vascular sensitivity to I/R (Figure 3E). Indeed, we reported that the hyperemic response, lost in HG animals, was restored in HG-Ex animals (Figure 2B,C), suggesting that endothelial function was protected during HG I/R by exercise. Thus, we next evaluated the impact of chronic exercise on the vascular response to Ach post I/R. In accordance with our previous results, we observed that exercise normalized the vascular response to ACh following I/R (+28% Rmax; HG-Ex vs. HG-Sed, *p* = 0.02, ES = 1.98; Figure 3E). This last result clearly explained the preserved arterial blood reflow observed at reperfusion in the previously trained HG group. Taken together, these results clearly demonstrated that the HG-dependent increased sensitivity to I/R of vascular tissues was counteracted by exercise training.

### 3.2. Impact of Hyperglycemia and Exercise Training on Arterial ROS Production and NO Availability

**Figure 4:**
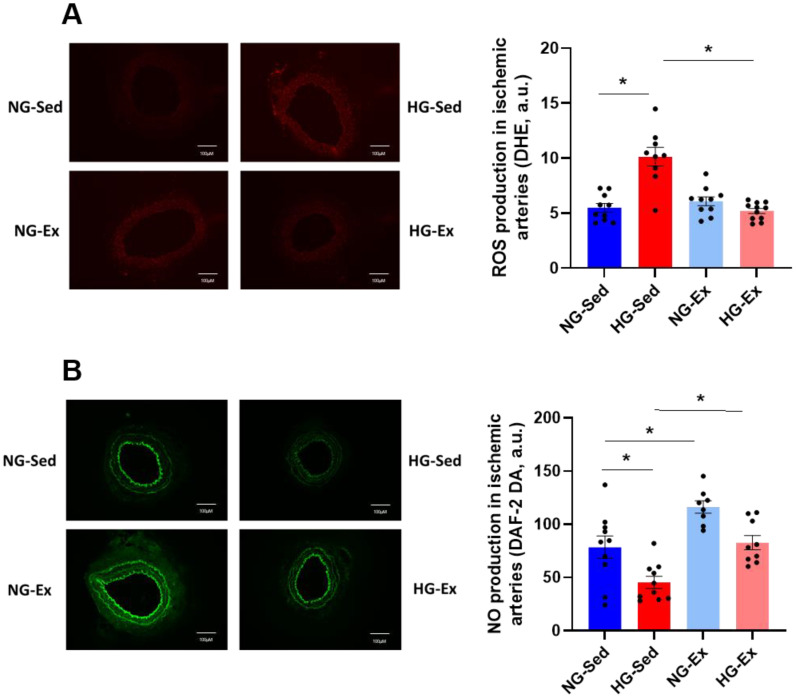
Normalization of reactive oxygen species and nitric oxide production contributes to the beneficial effects of exercise training on vascular function. (**A**) Reactive oxygen species production evaluated using dihydroethidium (DHE) incubation (5 min, 100 µM) on femoral artery rings following the ischemia-reperfusion protocol. Reactive oxygen species levels in each group were quantified using ImageJ software. (**B**) NO levels evaluated using the NO-sensitive fluorescent dye 4,5-diaminofluorescein diacetate (DAF-2, 60 min, 10 µM) on femoral artery rings following the ischemia-reperfusion protocol. NO levels in each group were quantified using ImageJ software. Fluorescence microscopy and digital image acquisition were carried out using an EVOS M5000 (Thermo Fischer Scientific, Waltham, MA, USA). Left panels represent illustrations of fluorescence photographs and right panels represent the quantification of reactive oxygen species and nitric oxide levels. *n* = 8–10 ischemic femoral rings from 4–5 rats per group. NG: normoglycemic; Sed: sedentary; HG: hyperglycemic; Ex: exercise training; A.U: arbitrary unit. *: *p* < 0.05. All values are expressed as the mean ± SEM. Comparisons of multiple experimental conditions were performed using analysis of variance (ANOVA) followed by Tukey’s post hoc test.

Considering that in the I/R environment, modulation of oxidative stress in the vascular wall may explain vascular alterations induced by HG and in part some protective effects of exercise training, we evaluated whether HG impacts the level of ROS production during postischemic reperfusion using the ROS-sensitive probe DHE. ROS production in vascular tissues was exacerbated in the HG-Sed group compared to the NG group (Figure 4A, NG-Sed vs. HG-Sed, *p* < 0.001, ES = 2.33). In line with the results obtained for perfusion, exercise training led to a normalization of ROS production in the HG-Ex group, with values significantly lower than those in the HG-Sed group (HG-Ex vs. HG-Sed, *p* < 0.001, ES = 2.59) and a similar range to those in the NG groups. In this context, we next evaluated whether HG impacted NO levels and if exercise training could preserve NO bioavailability using an NO-sensitive probe. Interestingly, while HG led to a significant drop in NO production in the sedentary group (NG-Sed vs. HG-Sed, *p* = 0.02, ES = 1.23), the HG-Ex group exhibited preserved NO levels in the postischemic femoral artery (Figure 4B, HG-Ex vs. HG-Sed, *p* = 0.007, ES = 1.97). Only a few effects of exercise were reported in our study in the NG condition; however, a positive impact of exercise training on NO level was observed. Indeed, DAF-2 fluorescence was significantly higher in NG-Ex than in NG-Sed post I/R arteries. (Figure 4B NG-Sed vs. NG-Ex, *p* = 0.009, ES = 1.43). Altogether, our data strongly support the beneficial effect of exercise training on ROS production and NO bioavailability observed post I/R in HG conditions.

## 4. Discussion

Despite the clinical importance of hyperglycemia-induced worsening of I/R injury, this is, to our knowledge, the first study to evaluate the protective effects of exercise training on the increased vascular sensitivity to I/R in HG. An original feature of this work is that this phenomenon was investigated in vivo in a territory with low energy metabolic activity to decipher specific vascular mechanisms. Our results support the hypothesis that exercise training is able to protect vascular function during I/R performed under HG conditions. Indeed, we reported for the first time, using in vivo experiments, that exercise training prevents the deleterious impact of I/R in HG on the vascular function through reduced ROS production and increased NO bioavailability.

Numerous studies have demonstrated the deleterious effects of HG on vascular endothelial function, both in the context of metabolic pathologies and in healthy subjects [2,11,38]. Furthermore, the aggravating effects of HG in ischemic pathology have been clearly demonstrated at the clinical level [3,4,5,6]. However, few studies have addressed the specific vascular effects induced by the presence of high blood glucose at the time of reperfusion. Several studies on the impact of HG on the vasculature during stroke have shown that HG can have a significant impact on the vascular system.

Indeed, to our knowledge, only a few studies have focused on the underlying vascular mechanisms that may explain this exacerbation of arterial lesions. However, as early as 1995, the importance of the effects of HG on the decrease in NO availability in the no-reflow mechanism observed following cerebral ischemia was demonstrated [25]. Subsequently, Fabian et al. [14] demonstrated in a model of I/R performed in the middle cerebral artery that HG negatively influenced the NO/ROS balance during I/R. As demonstrated in the present study, the authors described an increase in ROS production associated with a lower availability of NO in favor of ONOO^−^ formation. This functional uncoupling characterized by an increase in oxidative stress and a decrease in vasodilator factor resulted in a no-reflow phenomenon during the reperfusion period. Interestingly, in an original in vivo rat model, we were able to confirm that these alterations were indeed associated with the alteration of the endothelium-dependent response to hyperemia or ACh. This methodology allowed us to observe an impairment of the endothelium-dependent vasodilator response in the HG group that agrees with NO/ROS alterations. Taken together, our results strongly supported that HG-induced endothelial dysfunction is mediated by increased ROS production.

The main originality of our work was to evaluate the effects of a widely recognized cardiovascular protective strategy on the exacerbation of HG-induced I/R injury on the vasculature. Among protective strategies, a large number of studies have been able to demonstrate positive effects of exercise on vascular function [16,39]. More specifically, exercise training was proven to be able to limit endothelial vulnerability to I/R in both human [16,17] and rodent models [18,40]. However, different modalities of physical activity can be considered. Although intermittent exercise appears to be particularly interesting compared with continuous activity when endothelial function is studied in healthy subjects [41], this discrepancy is not always observed when the effects of different modalities of exercises are evaluated in the context of I/R whether in human [39] or animal [42] studies. When looking more specifically at the endothelial consequences of ischemia-reperfusion, the work of Thijssen et al. demonstrated a protective effect of physical activity independent of the type of exercise [43].

More generally, previous studies have shown that exercise training significantly improves endothelial function, assessed via flow mediated dilation in patients with heart failure, coronary artery disease or type 2 diabetes in human (see for review Pearson et Smart 2017) and animal models [44,45]. Yet, we previously reported that endothelial function plays a key role in the protective effects of exercise on cardiac ischemia/reperfusion [40]. In accordance with the hypothesis, our results highlighted the positive impact of exercise training performed at moderate intensity to counteract HG-induced increased sensitivity to I/R of vascular tissues. Indeed, we reported a restoration of vascular and muscular perfusion at the onset of reperfusion in the HG-Ex group. Interestingly, the in vivo vasoreactivity model used in this study managed to confirm the protective effect of exercise on endothelial function since the vascular response to ACh doses was normalized. This is a novel and important result to convince of the efficiency of exercise training to minimize HG-induced endothelial alterations during I/R. Interestingly, the positive impact of exercise training seems to be observed only in pathological conditions since perfusion and vasoreactivity were unchanged in NG-Ex compared to NG-Sed. Several mechanisms may explain the beneficial effects of exercise training on endothelial function in pathological conditions. First, chronic exercise training may increase antioxidant status and consequently reduce oxidative stress [46], which are exacerbated in HG groups. Yet, endothelial dysfunction has been associated with increased extracellular degradation of NO in the presence of ROS by the formation of ONOO^−^. Consequently, the blunting effect of exercise on ROS production should be responsible for increased NO availability. Our results are consistent with this hypothesis, since in our model, we reported both lower ROS production and higher NO availability in the vasculature of the previously trained HG group following I/R.

Altogether, our results confirm the deleterious effects of HG on vascular function during ischemia-reperfusion. Indeed, hyperglycemia was responsible for increased sensitivity of vascular function to I/R impairments. These impairments in vascular function post I/R were associated with exacerbated ROS production induced by HG, which consequently reduced NO bioavailability. Interestingly, for the first time, we provided evidence of the positive impact of chronic exercise training on protecting endothelial function during I/R in HG conditions. Indeed, chronic exercise seems to preserve vascular function from impairments induced by hyperglycemia during I/R through decreased ROS production and a subsequent enhancement of NO availability. These effects were able to prevent the no-reflow phenomenon induced by HG, which depends on an alteration of endothelial vasomotor function. Physical activity thus appears to be an efficient strategy to protect vascular function against the increased sensitivity to I/R induced by hyperglycemia. These results once again highlight the importance of physical activity as a strategy to limit susceptibility to cardiovascular pathologies.

## Data Availability

The data presented in this study are available on request from the corresponding author.
